# The Effect of Central Executive Load on Fourth and Sixth Graders’ Use of Arithmetic Strategies

**DOI:** 10.5334/pb.360

**Published:** 2017-07-13

**Authors:** Jiru Ai, Jia Yang, Tangzheng Zhang, Jiwei Si, Yaqiong Liu

**Affiliations:** 1 School of Psychology, Shandong Normal University, Jinan, CN

**Keywords:** central executive, computational estimation, strategy use, arithmetic, primary school children

## Abstract

In the present study, we set out to investigate whether and how central executive load constrains the strategies that children use during arithmetic processing. Using a dual-task paradigm accompanied by the choice/no-choice method, we tested 233 children (115 6^th^ graders, 118 4^th^ graders). Results showed that the impact of central executive load on reaction times and accuracy scores related to strategy use increased with the magnitude of the demands of the central executive, with central executive load playing an important role in strategy use. Sixth graders performed better than 4^th^ graders in the application of appropriate strategies. Children’s adaptability with respect to strategy choice was affected by the type and magnitude of the central executive load; children showed better adaptability under the no-load condition and the inconsistent/low load condition than under conditions with greater load. Grade level affected children’s adaptability with respect to strategy choice, with 6^th^ graders exhibiting significantly better performance than 4^th^ graders. These results confirm that the development of central executive skills contributes to children’s overall strategy use and adaptability. These findings have important implications for understanding the category specificity of central executive working memory in arithmetic cognition and the mechanisms of strategy development in childhood.

## Introduction

Arithmetic estimation, which includes computational estimation, magnitude estimation, and measurement estimation, is an important activity in mathematical cognition. Computational estimation involves a process of approximation in which individuals do not perform numerical calculation, but instead rely on their prior knowledge to provide a rough answer for a given problem. It requires the interaction of mental arithmetic, number concepts, and arithmetic skills (Si, 2002). Examining the mechanisms underlying computational estimation and its development may deepen our understanding of mathematical and general problem solving ability (Si, Yang, Jia, & Zhou, 2012). A substantial number of studies have shown that, when solving arithmetical problems, there were varieties of strategies which children choose to use (Lemaire & Lecacheur, 2002; Si & Zhang, 2003; Chen & Geng, 2005; Lemaire & Callies, 2009). Mental arithmetic refers to there is no external tools (such as a pen, calculator, etc.) in the process of arithmetic operations activities (Campbell, 2005). Arithmetic skills are, however, the individual ability of complete basic arithmetic (Imbo, 2007). These are two different concepts.

Children’s estimation strategies have been shown to be affected by arithmetic skills. Some of the most common estimation strategies among Chinese 6^th^ grade children were rounding to omit mantissa (Si & Zhang, 2003). Rounding has been investigated in many previous studies (Si, Yang, Jia, & Zhou, 2012; Lemaire, Arnaud & Lecacheur, 2004; Imbo, Duverne, & Lemaire, 2007; Lemaire & Lecacheur, 2010). In addition problems, rounding includes two different variants: rounding down (in which both of two operands are rounded down to the nearest whole tens; thus, 28 + 63 becomes 20 + 60 = 80) and rounding up (in which both of two operands are rounded up to the nearest whole tens; thus, 28 + 63 becomes 30 + 70 = 100). In the present study, these two strategies will be used to investigate children’s ability to solve arithmetic problems effectively and flexibly.

The most significant age-related changes in children’s arithmetic skills can be characterized in terms of strategy development (Lemaire, 2010). Changes in strategy choice may be affected by age-related changes pertaining to the central executive component of the working memory system (Lemaire, 2010; Lemaire & Lecacheur, 2011). However, to date, it remains unclear how age-related changes in the central executive system constrain the development of children’s arithmetic strategy use. Research has shown those children’s strategy use changes rapidly in the period between the 4^th^ and 6^th^ grades (Lemaire, Lecacheur, & Farioli, 2000). During the 4^th^, 5^th^ grades and 6^th^ grades time, speed of strategy use changes a lot (Lemaire, 2010). That is to say, the time between 4^th^ and 6^th^ grades is very important for the development of strategy use. This is also why we included these two grades in our study.

The central executive is the most complex component of working memory (Baddeley, 2010). It is an attention control system and its involvement is necessary for us to perform numerous higher-level cognitive activities such as chess playing. The central executive system can be divided into four distinct executive functions: memory updating, inhibition, switching, and dual-task coordination (Baddeley, 1996; Collette & Linden, 2002). Existing data have shown that various executive functions (memory updating, inhibition, switching, dual-task coordination) can affect children’s arithmetic performance and strategy use (Bull & Scerif, 2001). Researchers found links among dual-task coordination, switching, inhibition, updating, children’s strategy selection and execution (Chen & Wang, 2009). Although the various executive functions are separable, they are not completely independent; they have a common base in central executive working memory. Central executive working memory plays an important role in adults’ arithmetic behaviors (Baddeley, 1996). The central executive may affect strategy execution and strategy choice by participating in the carry operation involved in the process of calculation (Caviola, Mammarella, Cornoldi, & Lucangeli, 2012; Imbo, Vandierendonck, & Vergauwe, 2007). Most prior research has relied on measures of working memory span to explore the role of central executive load on the use of arithmetic cognitive strategies. For example, British children with low working memory spans were not able to select an efficient strategy when performing a multiplication task (Steel & Funnellf, 2001). In the latter study, retrieval was the fastest and least error-prone strategy, counting-in-series was the slowest and most error prone, and they also found that children mainly used mixed strategies. Working memory span affected children’s strategy distribution and implementation (Chen & Wang, 2009).

A perspective grounded in arithmetic strategy not only allows us to better understand the role of various factors and experimental situations, it also allows for a deeper understanding of individual differences in terms of skills, age, and cognitive abilities (Lemaire, 2010). Age was a strong determinant of children’s ability to apply the most appropriate strategy for a given problem (Lemaire & Lecacheur, 2011). Even when executive function was controlled for, children’s age still affected strategy choice. This is similar to previous findings that, with age increasing, children tend to apply more efficient and problem-adapted strategies (Lemaire & Callies, 2009; Barrouillet, Mignon, & Thevenot, 2008). In studying the link between the central executive and strategy use in children, Imbo and Vandierendonck used the choice/no choice method combined with a dual-task paradigm to investigate whether the application of retrieval, transforming, and counting strategies were influenced by central executive load among 4^th^, 5^th^, and 6^th^ grade children in estimations involving simple single-digit addition, and to explore any pertinent age-related changes. Regardless of load condition, they reported that children most frequently applied a retrieval strategy; age-related differences in strategy execution were not observed under working memory load (Imbo & Vandierendonck, 2007). It is worth noting that this study did not distinguish among different levels of load, relying on a binary distinction between conditions with and without load. If one were to implement a more powerful manipulation of the load factor, significant age-related effects might emerge. The present study, therefore, aimed to manipulate the load factor by combining load intensity and load type. An additional aim of our experimental design was to determine the degree of consistency between the main task and various types of secondary tasks. Thus, four different load levels were implemented consistent/high-load, consistent/low-load, inconsistent/high-load, and inconsistent/low-load. Previous strategy studies used the dual-task paradigm with an arithmetic task as the main tasks, with the secondary task typically non-numerical in nature. However, the consistency of the main and secondary tasks may have influenced children’s strategy use. This notion is supported by the findings of Logie, Gilhooly, and Wynn (1994), who observed that the type of secondary task influenced performance on a mental arithmetic task. In the present study, we used study materials adapted from Han and Kim (Han & Kim, 2004). We systematically manipulated central executive load by setting up conditions in which the primary and secondary tasks were consistent in type (i.e., a secondary task involving either digital recognition or a successive digit addition task) or inconsistent (i.e., a secondary task involving alphabetical ordering or letter recognition).

The choice/no choice method provides an unbiased estimate of individuals’ strategy choice (Siegler & Lemaire, 1997; Luwel, Onghena, Torbeyns, Schillemans, & Verschaffel, 2009). It involves two types of experimental conditions: the choice condition, under which subjects may freely choose which strategies they are going to use to solve problems, and the no-choice condition, under which subjects must use the specified strategy to solve all problems. The number of no-choice conditions should be equal to the number of possible strategies in the choice condition. As in our previous work (Si, Yang, Jia, & Zhou, 2012; Sun, Si, & Xu, 2012), the present study also implemented a best-choice condition, under which subjects were instructed to choose the most appropriate strategy to solve the given problem; this was done to further reveal the degree of flexibility of individuals’ strategy use. To prevent a general carry-over effect from the no-choice condition, the choice conditions were presented first. Additionally, the present research combined a dual-task paradigm with the choice/no choice method to examine children’s strategy use in a computational estimation task under different conditions of central executive load. The dual-task paradigm has been frequently implemented in both adult and child studies (Imbo & Vandierendonck, 2007; DeStefano & LeFevre, 2004).

Based on previous research, the present study aimed to examine children’s strategy performance (strategy selection and execution) in performing an addition estimation task by manipulating the complexity of the central executive load (i.e., load intensity and consistency of the main and secondary tasks). Under the high-load conditions, a successive digit addition task and an alphabetical ordering task were adopted as the secondary tasks; these primarily involve informational operations and updating. Under the low-load conditions, a digital recognition task and a letter recognition task were adopted as the secondary tasks, tasks that primarily involve information encoding and storage. Under the type-consistent conditions, the successive digit addition task and the digit recognition task were adopted as the secondary tasks, as both of these belong to the same category and compete for the same cognitive resources as the main task. Under the type-inconsistent conditions, the alphabetic ordering task and the letter recognition task were adopted as the secondary tasks. Compared with tasks that fall in the same category, tasks belonging to different categories seldom compete for the same resources. By comparing load intensity and load type, we hoped to observe any pertinent differences in the influence of the central executive load on children’s strategy use as a function of increasing age, and to further elucidate the linkage between the central executive and cognitive strategy use. We assumed that under the condition of high level central executive load, both of their performance get worse compared with no load or low level load. And those 4th grade children performed worse.

Arithmetic skills are the ability of complete basic arithmetic. It has an obvious influence on strategy use and strongly influence strategy choice (Thevenot, Fanget, & Fayol, 2007; Imbo, Vandierendonck & Rosseel, 2007). A number of researchers have used arithmetic skill as a covariate to examine individuals’ strategy use (Imbo & LeFevre, 2010; Barrouillet & Lépine, 2005; Miyake, Friedman, Emerson, Witzki, Howerter, & Wager, 2000). In the present study, we controlled for arithmetic skill and examined whether the central executive working memory load has a separate influence on children’s strategy use over time, independent of arithmetic skill.

## Method

### Participants

A total of 255 children from two ordinary primary schools in China (including 130 4^th^ graders and 125 6^th^ graders) were selected. All participants were required first to complete the arithmetic skills test, and then to simultaneously complete the addition estimation task and the secondary task (if any). Based on this testing, 233 subjects with normal eyesight or corrected normal eyesight were retained in the final sample (113 boys and 120 girls; 118 4^th^ graders and 115 6^th^ graders; average age, 10.63 ± 1.27 years).

### Design

In this study, “consistent” means both the main task and the secondary task involve digital operations. Correspondingly, “inconsistent” means two tasks involve different operations (main task involving numbers, secondary task involving letters). A 5 (load situation: consistent/high load, inconsistent/high load, consistent/low load, inconsistent/low load, and no load) × 3 (strategy use condition: best choice (C1), rounding up (C2), and rounding down (C3)) × 2 (grade level: 4 and 6) mixed experimental design was used. Load and grade level were implemented as between-subjects variables (the participants in each group are shown in Table 1), and strategy use condition as a within-subject variable. The task was presented using a dual-task paradigm involving arithmetic estimation as the main task and number or letter judgment as the secondary task.

**Table 1:** Numbers of participants allocated in different load situations.

	Consistent–high load	Consistent– low load	Inconsistent– high load	Inconsistent–low load	No load	Total

6^th^ grade	22	19	25	23	26	115
4^th^ grade	25	23	20	25	25	118
Total	47	42	45	48	51	233

### Materials

#### Arithmetic tests

The French Kit test was adopted (French, Ekstrom, & Price, 1963). The test contains two subtests; one involves complex addition with three addends, and the other including mixed subtraction and multiplication problems involving two subtrahends or two multipliers. Each subtest is divided into two parts; each part contains 60 questions with a 2-minute answer time. The number of correct responses corresponds to the test score. Many previous studies have adopted this tool to measure individuals’ arithmetic skills (Thevenot, Fanget, & Fayol, 2007; Ai, Zhang, Si, Lu, & Zhang, 2016; Huang, Feng, Si, Zhang, & Wang, 2016).

#### Main task

Thirty two-digit addition estimation problems (for example, 76 + 42) were used in the main task, including 15 rounding-down problems (in which the rounding-down strategy was required for estimation, such that 51 + 78 becomes 50 + 70 = 120), 15 rounding-up problems (in which the rounding-up strategy was required for estimation; thus, 74 + 69 becomes 80 + 70 = 150). In half of the problems, the unit digit of the first addend was less than 5, and the unit digit of the second addend was greater than 5. The other half of the problems was structured in the opposite manner. In half of the problems, the greater addend was on the left (e.g., 86 + 52), and in the remaining half of the problems, the greater addend was on the right (e.g., 43 + 86). In addition to the above constraints (Campbell, 2005), addition problems used in arithmetic cognition research should exclude situations in which: (1) the unit digit of an addend corresponds to zero or five; (2) two addends in the same location (units, tens) are repeated in the same problem (e.g., 23 + 63, 24 + 26); (3) units and tens are repeated in one addend (e.g., 66 + 31); and (4) two identical addends, but with the positions reversed, are used in two separate problems (e.g., if 32 + 47 occurs in one problem, 47 + 32 should not be permitted to occur in another problem). Problems in the present study were excluded based on those constraints.

#### Secondary task materials

Materials adapted from Han and Kim were used (Si, Yang, Jia, & Zhou, 2012; Han & Kim, 2004). These materials were divided into four types: (1) the consistent/high-load task, in which a three-digit number was randomly presented at the center of the display; participants were required to successively add the three numbers and to report their results orally; (2) the consistent/low-load task, in which participants were required to remember a random six-digit number string presented on the screen and to determine whether a subsequently presented number had appeared in this number string; (3) the inconsistent/high-load task, in which three random alphabet letters were presented at the center of the screen, and participants were required to repeat these letters in alphabetical order; and (4) the inconsistent/low-load task, in which three random alphabet letters were presented at the center of the screen, and participants were merely required to retain these letters in memory, and then to judge whether the letters subsequently presented had been previously presented.

### Procedure

The experiment was divided into three parts: the best-choice condition (C1), under which participants were instructed to choose between two given strategies (rounding up and rounding down) to arrive at an answer that approximated the accurate sum; the no-choice/rounding-up condition (C2), under which participants were instructed to use only the rounding-up strategy to arrive at their estimates; and the no-choice/rounding-down condition (C3), under which subjects were instructed to apply only the rounding-down strategy to estimate the sum. Participants were instructed to type in their responses as quickly and accurately as possible.

The rounding-up strategy means rounding the two addends up to their nearest tens (73 + 49 → 80 + 50, the answer is 130); The rounding-down strategy involved adjusting both addends down to their nearest tens (73 + 49 → 70 + 40, the answer is 110). A mixed strategy in which one addend was rounded up and the other was rounded down was not permitted throughout the entire experiment.

To avoid any influence of the no-choice conditions on the execution of strategies in the choice condition, we first tested all participants with stimuli from C1 (the best-choice condition), followed by C2 and C3. The interval between any two conditions was 5 minutes, each of 30 trials.

Each of the participants completed 10 practice trials to become familiarized with the experimental procedure and tasks before the formal experiment.

**Figure 1:**
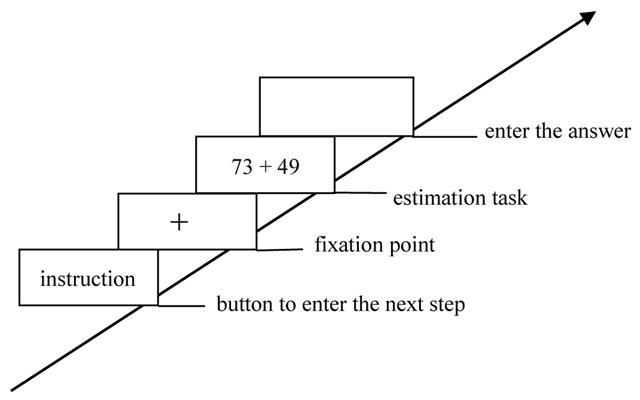
Flow chart of no-load condition.

**Figure 2:**
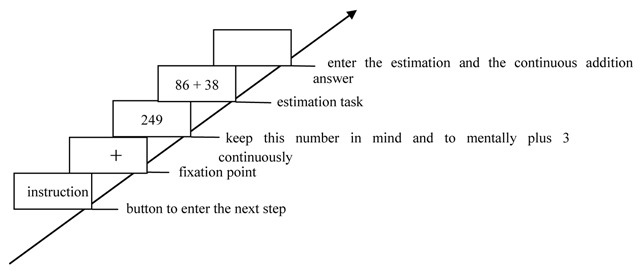
Flow chart of consistent/high-load condition.

**Figure 3:**
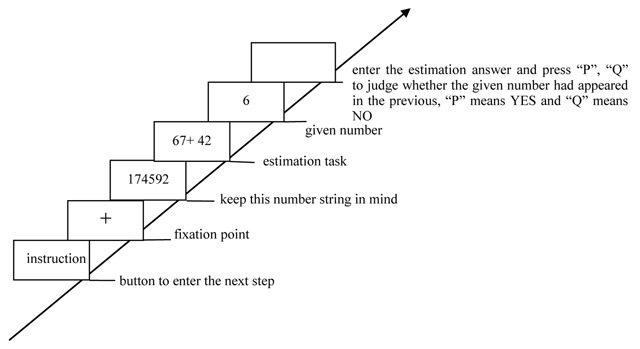
Flow chart of consistent/low-load condition.

**Figure 4:**
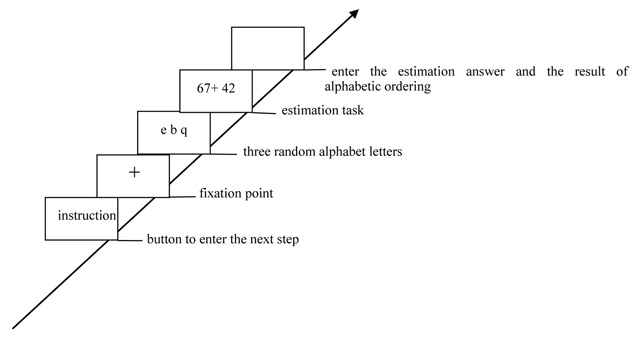
Flow chart of inconsistent/high-load condition.

**Figure 5:**
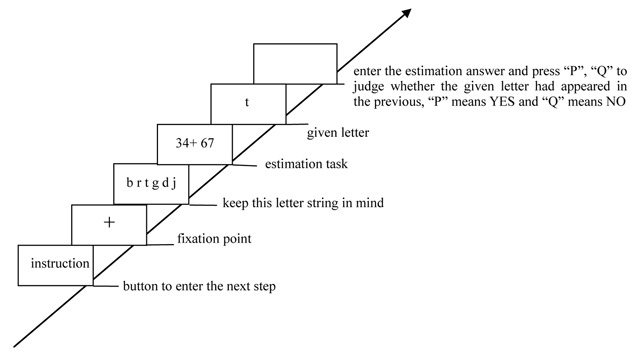
Flow chart of inconsistent/low-load condition.

In no-load condition, participants were only required to complete the estimation task (Figure 1). In the consistent/high-load condition, participants were required to simultaneously perform the successive digit addition task (keep three-digit number in mind and to mentally add 3 continuously) and the estimation task (Figure 2). In the consistent/low-load condition, participants should simultaneously perform the digit recognition task (keep six-digit number string in mind and press a key to indicate whether that given number had appeared in the previous six-digit number string) and the estimation task (Figure 3). In the inconsistent/high-load condition, participants were required to simultaneously perform the alphabetical sorting task (mentally alphabetize the three random alphabet letters and upon pressing “Enter,” they were to immediately type in the result of their alphabetic ordering) and the estimation task (Figure 4). In the inconsistent/low-load condition, participants were required to simultaneously perform the letter recognition (simply retained the presented letters in memory and press a key to indicate whether that given letter had appeared previously) and estimation tasks (Figure 5).

## Results

### Strategy execution

Reaction times and accuracy scores obtained under the no-choice rounding-up condition provide an unbiased estimate of the execution of the rounding-up strategy; similarly, the execution of the rounding-down strategy is reflected by these measures under the no-choice rounding-down condition. Reaction times for responses in which participants failed to apply the given strategy were excluded.

#### Reaction time

Using the arithmetic skill score as a covariate and reaction time for the estimation task in C2 and C3 as the dependent variable, we conducted a 2 (strategy use condition) × 5 (load situation) × 2 (grade level) repeated-measures analysis of variance. Results revealed a significant main effect of arithmetic skill (*F*_(1,222)_ = 38.11, *p* < 0.05, *η^2^* = 0.147). This indicates that controlling for arithmetic skills is meaningful. There was also a significant main effect of strategy use condition (*F*_(1,222)_ = 33.71, *p* < 0.05, *η^2^* = 0.132). Specifically, execution times for the rounding-up strategy were significantly longer than were those for the rounding-down strategy, suggesting that the rounding-up strategy is more complex than the rounding-down strategy. Load situation also exerted a significant main effect, indicating that the nature of the central executive load influenced reaction times for strategy execution (*F*_(4,222)_ = 10.38, *p* < 0.05, *η^2^* = 0.158). Post hoc tests using the least significant difference (LSD) procedure revealed that the following conditions differed significantly from the no-load condition: the consistent/high-load condition (*p* < 0.001), the consistent/low-load condition (*p* = 0.018), the inconsistent/high-load condition (*p* < 0.001), and the inconsistent/low-load condition (*p* = 0.020). Furthermore, the consistent/high-load condition differed significantly from the inconsistent/low-load (*p* = 0.022) and the consistent/low-load conditions (*p* = 0.038). Additionally, the inconsistent/high-load condition differed significantly from the consistent/low-load (*p* = 0.001), and the inconsistent/low-load conditions (*p* = 0.001). However, there were no significant differences between the inconsistent/high-load and the consistent/high-load conditions (*p* = 0.216) or between the inconsistent/low-load and the consistent/low-load conditions (*p* = 0.892). The above results indicate that response times reflecting children’s strategy execution increase as the intensity of the load rises but that load type does not affect reaction time. Additionally, there was a significant main effect of grade level (*F*_(1,222)_ = 46.03, *p* < 0.05, *η^2^* = 0.172). Specifically, the reaction times of 6^th^ graders were faster than those of 4^th^ graders.

**Figure 6:**
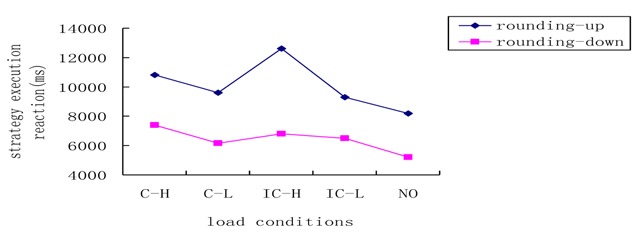
Reaction time of strategy execution in different strategy use conditions and load situations. C–H: consistent-high load; C–L: consistent-low load; IC–H: inconsistent-high load; IC–L; inconsistent-low load; NO: no load.

A noteworthy interaction emerged between strategy use condition and load situation (*F*_(4,222)_ = 4.72, *p* < 0.05, *η^2^* = 0.078), as shown in Figure 6. This suggests that the execution of rounding-up and rounding-down strategies is influenced by central executive load. Further simple effects analyses revealed that under the no-choice and rounding-up conditions, the inconsistent/high-load condition differed significantly from the consistent/high-load (*p* = 0.015), the consistent/low-load (*p* < 0.001), and the inconsistent/low-load conditions (*p* < 0.001). Additionally, the difference between the consistent/high-load and the inconsistent/low-load conditions was not significant (*p* = 0.052). The no-load condition did not differ significantly from the consistent/low-load (*p* = 0.078), or the inconsistent/low-load condition (*p* = 0.118). Under the no-choice and rounding-down strategy conditions, no differences emerged among the inconsistent/high-load, consistent/high-load, consistent/low-load, and the inconsistent/low-load conditions. This indicates that compared with the rounding-down strategy under the no-choice condition, reaction times reflecting children’s execution of the rounding-up strategy under the no-choice condition showed greater sensitivity to changes in load complexity. It appears that the central executive load may exert a stronger influence on the execution of complex than that of simpler strategies. Additionally, the inconsistent/high load condition seems to lead to detrimental effects when children use a complex strategy (rouding up). The reason for this result may be that, for China’s primary school students, alphabetical sorting is relatively difficult especially when they must also have to complete the estimate task.

**Figure 7:**
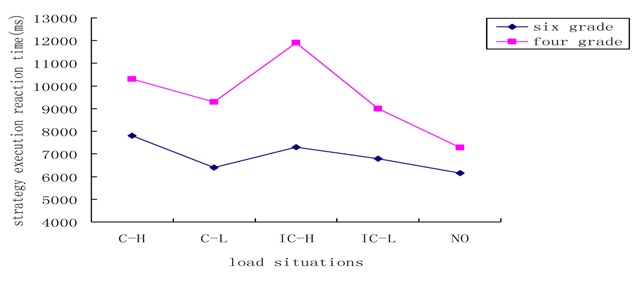
Reaction time of strategy execution in different grades and load situations. C–H: consistent-high load; C–L: consistent-low load; IC–H: inconsistent-high load; IC–L; inconsistent-low load; NO: no load.

As can be seen in Figure 7, there was a significant interaction between grade level and load condition (*F*_(4,222)_ = 2.75, *p* < 0.05, *η^2^* = 0.047). The two grade levels exhibited different performance across the different load situations. Simple effects tests revealed that for the 6^th^ graders, there were no significant differences between any of the following: the consistent/high-load and inconsistent/high-load conditions (*p* = 0.630), the consistent/low-load and the inconsistent/low-load conditions (*p* = 0.583), the consistent/low-load and the no-load conditions (*p* = 0.809), and the inconsistent/low-load and no-load conditions (*p* = 0.401). For the 6^th^ graders, strategy execution time was affected by load intensity. However, low load had no effect on strategy execution time; it appears that it is only when the central executive system is sufficiently taxed that an impact on 6^th^ graders’ strategy execution time was observed, slowing down response times. In contrast to load intensity, the influence of load type on strategy execution time at this grade level was not substantial. The inconsistent/high load condition seems to lead to detrimental effects in young children. Similar to the results before, for younger children, alphabetical ordering is worse than for 6^th^ graders. Therefore, they are more likely to be affected.

A different pattern emerged for 4^th^ graders. The difference between the consistent/high-load and inconsistent/high-load conditions was not significant (*p* = 0.077), nor was the difference between the consistent/low-load and the inconsistent/low-load conditions (*p* = 0.554) or that between the inconsistent/low-load and the no-load conditions (*p* = 0.059). However, the inconsistent/low-load condition differed significantly from the consistent/low-load condition (*p* = 0.001) and from the inconsistent/high-load condition (*p* = 0.005). Furthermore, the difference between the consistent/low-load and the no-load conditions was also significant (*p* = 0.015). From these results, it can be inferred that for younger children, there is a greater likelihood that the central executive load will interfere with strategy execution. Under the low-load conditions, the influence of load type was significant, with consistent tasks exerting greater interference compared with inconsistent tasks. Even low levels of central executive load affected strategy execution among 4^th^ graders, whereas low load had little effect on 6^th^ graders.

No significant interaction was found between strategy use and grade level (*F*_(4,222)_ = 1.86, *p* > 0.05, *η^2^* = 0.008). Sixth graders showed similar advantages in terms of speed of responding relative to 4^th^ graders under both the rounding-up and rounding-down strategy conditions. The three-way interaction of strategy use condition by grade level by load situation was not significant (*F*_(4,222)_ = 1.34, *p* > 0.05, *η^2^* = 0.023).

#### Accuracy

Using participants’ accuracy scores for the estimation task under conditions C2 and C3 as the dependent variable and arithmetic skill as the covariate, we conducted a 2 (strategy use condition) × 5 (load situation) × 2 (grade level) repeated-measures analysis of variance. The results yielded no significant main effect of arithmetic skill (*F*_(1,222)_ = 0.24, *p* > 0.05, *η^2^* = 0.001), indicating that arithmetic skill did not affect the accuracy of strategy execution. Likewise, there was no significant main effect of strategy use condition (*F*_(1,222)_ = 1.95, *p* > 0.05, *η^2^* = 0.009), showing that there was no significant difference in accuracy between the rounding-up and rounding-down strategies. However, there was a significant main effect of grade level (*F*_(1,222)_ = 12.76, *p* < 0.05, *η^2^* = 0.054), with 6^th^ graders exhibiting better accuracy than 4^th^ graders. There was also a main effect of the load situation (*F*_(4,222)_ = 5.99, *p* < 0.05, *η^2^* = 0.097), demonstrating an impact of the central executive load on accuracy in the estimation task. Furthermore, a Bonferroni test using LSD revealed robust differences between the no-load condition and the other load conditions, namely, the consistent/high-load condition (*p* < .001), the consistent/low-load condition (*p* = 0.001), inconsistent/high-load condition (*p* < .001), and the inconsistent/low-load condition (*p* = 0.010) (note that all the p-values were considerably below the 0.05 threshold). However, there were no significant differences among the various load conditions. These results suggest that the presence of any degree or type of central executive load has an impact on the accuracy of children’s estimation performance and that this impact can be seen at various ages.

There was a significant interaction between strategy use condition and grade level (*F*_(4,222)_ = 6.82, *p* < 0.05, *η^2^* = 0.030). A significant difference emerged between the 4^th^ (*M* = 0.869, *SD* = 0.012) and 6^th^ graders (*M* = 0.967, *SD* = 0.012) under the no-choice and rounding-up conditions. However, the no-choice and rounding-down conditions yielded no significant differences between 4^th^ (*M* = 0.945, *SD* = 0.007) and 6^th^ graders (*M* = 0.936, *SD* = 0.007). These results indicate that strategy complexity affects children’s estimation accuracy. The period between the 4^th^ and 6^th^ grades appears to be an important period in terms of changes in performance accuracy related to strategy execution. There were no significant interactions between strategy use condition and load situation (*F*_(4,222)_ = 1.02, *p* > 0.05, *η^2^* = 0.018), between grade level and load situation (*F*_(4,222)_ = 0.99, *p* > 0.05, *η^2^* = 0.018), or among strategy use condition, grade level, and load situation (*F*_(4,222)_ = 0.93, *p* > 0.05, *η^2^* = 0.016).

### Strategy choice

Reaction time and accuracy scores of subjects under the best-choice condition (C1) reflect their strategy choices. We excluded reaction times for trials in which participants failed to apply one of the two targeted strategies (rounding up or rounding down). The accuracy score for each rounding-up condition was computed as the number of trials in which participants correctly applied the rounding-up strategy when it was optimal to do so divided by the total number of trials in which the rounding-up strategy was used. Similarly, the accuracy score for each rounding-down condition equaled the number of trials in which participants correctly applied the rounding-down strategy when it was optimal to do so divided by the total number of trials in which the rounding-down strategy was used.

#### Reaction time

Using participants’ reaction times for executing rounding-up and rounding-down strategies under the best-choice condition (C1) as the dependent variable and arithmetic skills as the covariate, we conducted a 2 (strategy type) × 5 (load situation) × 2 (grade level) repeated measures analysis of variance. The results revealed a robust main effect of arithmetic skill, *F*_(1,222)_ = 16.73, *p* < 0.05, *η^2^* = 0.070, indicating that it was meaningful to considering arithmetic skill as a covariate. There was also a significant main effect of strategy type, *F*_(1,222)_ = 5.32, *p* < 0.05, *η^2^* = 0.023. Specifically, the rounding-down strategy yielded significantly faster reaction times (RTs) than the rounding-up strategy. Moreover, there was also a significant main effect of load situation, *F*_(4,222)_ = 3.52, *p* < 0.05, *η^2^* = 0.060, suggesting that the nature of the central executive load affected RTs when strategy choice was permitted. Further post-hoc analyses using LSD indicated that the no-load condition differed significantly from the consistent/high-load condition (*p* = 0.002), the consistent/low-load condition (*p* = 0.011), the inconsistent/high-load condition (*p* = 0.002), and the inconsistent/low-load condition (*p* = 0.008), with all differences exhibiting *p*-values < 0.05. Hence, it appears that the presence of any central executive load may result in slower RTs under a condition of strategy choice. The process of strategy selection involves sub processes pertaining to both selection and execution; compared with strategy execution, strategy selection requires greater involvement of the central executive component. Thus, performance under even minimal load conditions was understandably worse than that under the no-load condition. There was no significant main effect of grade level (*F*_(1,222)_ = 3.50, *p* > 0.05, *η^2^* = 0.016), and no significant interaction of grade level × load situation (*F*_(4,222)_ = 1.92, *p* > 0.05, *η^2^* = 0.033), grade level × strategy type (*F*_(1,222)_ = 0.30, *p* > 0.05, *η^2^* = 0.001), load situation × strategy type (*F*_(4,222)_ = 0.69, *p* > 0.05, *η^2^* = 0.012), or load situation × grade level × strategy type (*F*_(4,222)_ = 0.31, *p* > 0.05, *η^2^* = 0.006).

#### Accuracy

We conducted a 2 (strategy type) × 5 (load situation) × 2 (grade level) repeated-measures analysis of variance in which the dependent variable was accuracy scores for rounding-up and rounding-down strategies on the estimation task under the best-choice condition (C1), and arithmetic skill was the covariate. The results revealed no significant main effect of arithmetic skill (*F*_(1,222)_ = 0.57, *p* > 0.05, *η^2^* = 0.003), indicating that the accuracy of children’s strategy choice was not affected by their level of arithmetic skill. The load situation did yield a significant main effect (*F*_(4,222)_ = 3.29, *p* < 0.05, *η^2^* = 0.056), indicating that the central executive load interfered with the accuracy of strategy selection. Post hoc tests using LSD revealed that the no-load condition differed significantly from the consistent/high-load (*p* = 0.001), consistent/low-load (*p* = 0.027), inconsistent/high-load (*p* = 0.013), and the inconsistent/low-load condition (*p* = 0.040), with all *p*-values < 0.05. This finding suggests that any type of central executive load may affect the accuracy of strategy choice. There was also a significant main effect of grade level (*F*_(1,222)_ = 14.18, *p* < 0.05, *η^2^* = 0.060), suggesting that the strategy accuracy of the 6^th^ graders was significantly higher than that of the 4^th^ graders. However, the main effect of strategy type was very weak (*F*_(1,222)_ = 0.28, *p* > 0.05, *η^2^* = 0.001). All of the interactions were non-significant, including load situation by grade level (*F*_(4,222)_ = 1.97, *p* > 0.05, *η^2^* = 0.034), grade level by strategy type (*F*_(1,222)_ = 0.04, *p* > 0.05, *η^2^* = 0.000), load situation by strategy type (*F*_(4,222)_ = 2.08, *p* > 0.05, *η^2^* = 0.036), and load situation by grade by strategy type (*F*_(4,222)_ = 0.49, *p* > 0.05, *η^2^* = 0.009).

### Adaptability of strategy choice

**Figure 8:**
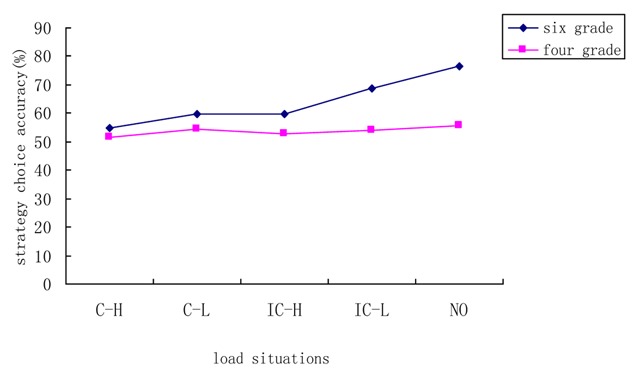
Adaptability of strategy choice in 4^th^ and sixth- grade children under different load situations. C–H: consistent-high load; C–L: consistent-low load; IC–H: inconsistent-high load; IC–L; inconsistent-low load; NO: no load.

We defined the adaptability of strategy choice in terms of the ability to choose the strategy that most closely approximated the accurate answer to the addition problem (Lemaire, Arnaud & Lecacheur, 2004). adaptability in the present study was reflected in accuracy scores for strategy choice under the best-choice condition (C1). Accuracy scores referred to the percentage of correct estimation (the number of right estimate problems divided by the total number of problems). Because in C1, participants can perform the estimation task correctly only by choosing the accurate strategy. Using these accuracy scores in condition C1 as the dependent variable and arithmetic skill as the covariate, we conducted a 5 (load situation) × 2 (grade level) analysis of variance. The results revealed no significant main effect of arithmetic skill (*F*_(1,222)_ = 0.27, *p* > 0.05, *η^2^* = 0.001), indicating that strategy adaptability was not affected by children’s level of arithmetic skill. There was a significant main effect of load situation, *F*_(4,222)_ = 4.14, *p* < 0.05, *η^2^* = 0.069, showing that the central executive load affected children’s strategy adaptability. Further post hoc tests using LSD revealed that the no-load condition differed significantly from the consistent/high-load condition (*p* < .001), the consistent/low-load condition (*p* = 0.015), and the inconsistent/high-load condition (*p* = 0.005). Furthermore, the differences between the consistent/high-load condition and the inconsistent/low-load was significant (*p* = 0.022), whereas that between the inconsistent/low-load condition and the no-load condition was not (*p* = 0.164). These results suggest that both load type and intensity affected the adaptability of children’s strategy choice. Children displayed a degree of adaptability similar to that under the no-load condition only in situations with both low load intensity and low demands due to load type. There was a significant main effect of grade level, *F*_(1,222)_ = 13.41, *p* < 0.05, *η^2^* = 0.057, suggesting that the strategy adaptability of 6^th^ graders was considerably higher than that of 4^th^ graders, implying that the period between the 4^th^ and 6^th^ grades may be a developmentally important period with regard to this particular skill. However, there was no significant interaction of grade level by load situation (*F*_(4,222)_ = 2.20, *p* > 0.05, *η^2^* = 0.038). The adaptability of strategy choice among 4^th^ graders was lower than that among 6^th^ graders under the various load situations. The adaptability of children’s strategy choices displayed a relatively consistent trend across all five load situations (see Figure 8). These results indicate that both the central executive load type and intensity affect individuals’ adaptability of strategy choice in childhood.

## Discussion

This study examined the impacts of various loads on central executive functioning in children’s estimation strategies at different ages. Results showed that the central executive load affected children’s strategy performance. The heavier the load is, the greater the impact on children and 4^th^ grade children were more susceptible.

### Effects of central executive load on children’s strategy execution at different ages

The presence of any central executive load resulted in interference, as seen in the overall decline of strategy execution efficiency (reaction times and accuracy scores). To some degree, this is consistent with Wang and Chen’s study (Wang & Chen, 2006). Specifically, we found that as the magnitude of the load increased, reaction times for strategy execution became longer and that children’s accuracy in strategy execution declined, even at relatively low levels of load complexity. Our findings differ from those of Imbo and Vandierendonck (2007), who found no impact of load on the accuracy of strategy execution, but we did find an interaction between children’s age and working memory load. One possible explanation for the discrepant results may be that Imbo and Vandierendonck only distinguished between the presence and absence of cognitive load, disregarding variations in load intensity. Another explanation may lie in the difficulty of the main task and the relationship between the primary and secondary tasks. Imbo and Vandierendonck used a one-digit addition estimation problem (involving addends between 2 and 9) as the primary task. Such a task would be very easy for children in grades 4 and 6. Moreover, their secondary task, CRT (a pitch judgment task) had minimal association with the primary task, resulting in very limited competition for children’s working memory resources. The present study made finer distinctions with respect to the type and intensity of the central executive load. The level of load complexity was more difficult overall, and the main task involved the more demanding two-digit addition estimation task.

The specific pattern of these effects appears to change with age. Fourth grade children were more sensitive to slight increases in central executive load due to their limited working memory resources, with the result that their performance on the main task deteriorated relative to the single task (no-load condition) even under minimal load. When working memory resources are not already subject to heavy strain, the competition between the category-consistent primary and secondary tasks is considerably higher than the competition that arises in category-inconsistent situations. Accordingly, we found that 6^th^ grade children were able to complete dual tasks with ease when the secondary task was inconsistent, creating less competition for resources.

Si, Yang, Jia, and Zhou (2012) found that adults were able to accomplish simple dual tasks very well. However, in a complex dual task requiring more resources, the impact of different types of loads emerged. Thus, there are age-related differences in the effects of load complexity on strategy use. The higher the central executive load is, the greater is the demand for cognitive resources, and the longer it takes to perform a primary task, which is in accordance with the theoretical perspective of Case (1985) and Towse and Hitch (1997). For younger children under low-load situations, the secondary task, which involves the encoding and storage of information, creates substantial competition for resources, resulting in differences in strategy execution across different task categories. The presence of such effects under conditions of low load suggests that children at this stage are in the midst of a period of development with respect to working memory encoding and storage. However, older children are only affected by load intensity and not by load type, suggesting a transition period related to the coordination of storage and processing. To some degree, these results support multiple resource sharing models. The central executive restricts the storage, processing, and operations that can be performed by cognitive resources when there is limited working memory capacity.

Increasing age resulted in a gradual improvement in the speed and accuracy of children’s strategy execution under conditions of central executive load. Both reaction times and accuracy scores for strategy execution showed considerably stronger performance among 6^th^ graders than among 4^th^ graders. The development of working memory resources and executive functions may play an important role in age-related differences in strategy use (Lemaire & Lecacheur, 2011; Hodzik & Lemaire, 2011). Working memory capacity develops with age. It exhibits particularly rapid development before 8 years of age, slower development between 8 and 20 years of age, and then begins to decline after the age of 20 (Li, Bai, & Shen, 2006).

Our study further confirms that the influence of central executive load on strategy execution changes with age. Strategy execution among 6^th^ grade children is superior relative to that that among 4^th^ graders, and accuracy improves for the more complex rounding-up strategy. This supports the notion that the period between grades 4 and 6 represents a key developmental period with respect to strategy execution in estimation tasks (Lemaire, Lecacheur, & Farioli, 2000; Lemaire & Lecacheur, 2002). The results indicate that increasing age and experience are associated with greater accuracy and faster reaction times among Chinese children performing two-digit addition estimation tasks and that the execution of rounding-up and rounding-down strategies shows a development progression, with particularly strong developmental effects observed for the rounding-down strategy.

### Effects of central executive load on children’s strategy choice and adaptability at different ages

The presence of the central executive load of any type or intensity affected children’s reaction times and accuracy scores when strategy choice was involved. Strategy use involves processes related to both selection and execution. Of the two processes, strategy choice requires greater central executive capacities and is more easily affected by the central executive load than strategy execution. However, Imbo and Vandierendonck’s (2007) findings are at odds with the above generalization, possibly because their experimental study relied on different measures than ours. Imbo and Vandierendonck adopted the frequency of retrieval, switching, and counting as strategy indicators, whereas the present study used reaction times and accuracy of rounding-up and rounding-down strategies. Under conditions of strategy choice, children gravitate toward the excessive use of simple or repeated strategies, as evidenced by the repeated-strategy effect and strategy-switching cost observed particularly under load situations (Lemaire & Lecacheur, 2010). This tendency is linked to children’s imperfect development of executive function, particularly the imperfect development of abilities related to inhibition and switching. Previous studies have confirmed that executive function plays a role in age-related differences in strategy choice (Lemaire & Lecacheur, 2010; Hodzik & Lemaire, 2011). The rapid growth of children’s executive function occurs between 7 and 9 years and between 10 and 12 years of age; however, developmental patterns for inhibiting, switching, updating, and other functions show distinct profiles (Wang, Chen, & Zhong, 2009). As is apparent under conditions of central executive load, children from grades 4 to 6 are in a period of rapidly developing executive function and lack adequate working memory resources to suppress previous strategies and to select and switch to more effective strategies. Age can be seen to affect children’s strategy selection. Lemaire and Lecacheur (2011) found that, even when inhibition and cognitive flexibility were controlled for, children’s age still played a significant role in strategy choice (Lemaire & Lecacheur, 2011). Hodzik and Lemaire also found that after statistically controlling for the effects of inhibition and switching, the effect of children’s age on strategy choice remained significant (Hodzik & Lemaire, 2011). In our study, under conditions of central executive load, the accuracy of strategy choice among 6^th^ graders increased relative to that of 4^th^ graders, with the older children choosing and executing more effective strategies, including the more complex rounding-up strategy, to solve problems. This indicates that changes in children’s strategy development are influenced by age and by changes in the central executive function (Lemaire & Lecacheur, 2010; Lemaire, 2010). It might be appropriate to acknowledge that also the arithmetic task becomes more automatized in 6^th^ grade, which also might influence these results.

We observed effects of various types and intensities of load on the adaptability of children’s strategy choice. In situations with no load or inconsistent/low load, children exhibited greater accuracy and better adaptability in their strategy choices. Previous findings have shown that the presence of any load affected the adaptability of adults’ strategy choices (Si, Yang, Jia, & Zhou, 2012; Imbo & LeFevre, 2009; Imbo & LeFevre, 2011); however, there are notable age-related differences between children and adults. Developing children are sensitive to the effects of type and intensity of load, and the presence of any central executive load has a measurable effect on their adaptability. Compared with adults, children generally exhibit lesser adaptability in strategy choice and may often fail to choose the strategy that arrives at the best estimated value. Sixth grade children are increasingly able to choose the best strategy, and their strategy adaptability is superior to that of 4^th^ grade children. However, children’s adaptability with respect to strategy choice remains in need of further development, as evidenced by previous findings (Lemaire & Callies, 2009; Waters & Schneider, 2010; Barrouillet, Mignon, & Thevenot, 2008). An additional finding of our study was that Chinese children’s strategy adaptability was not affected by arithmetic skill. This finding is similar with Imbo and LeFevre (Imbo & LeFevre, 2009; Imbo & LeFevre, 2011). The arithmetic skills of Chinese participants are very high, and yet their adaptability is vulnerable to effects of the central executive load. Therefore, arithmetic skills cannot be responsible for the differences observed among Chinese children with respect to strategy adaptability. According to Imbo and LeFevre, the adaptability of Chinese participants’ strategy choices was lower than that of their peers in North America and Europe, perhaps due to traditional style of Chinese instruction (Imbo & LeFevre, 2011). It would be useful to implement and study training regimes for working memory with the aim of extending the universality of the above findings. Additionally, the approximate number system (ANS) is the basis for human mastery of arithmetic skills (Jiang, 2011). It remains to be seen whether this approximate number system plays a regulatory role with respect to the impact of the central executive load on strategy adaptability.

### Summary

Our results indicate that among 4^th^ graders, the presence of any degree of central executive load affected strategy use. By the 6^th^ grade, the impact of low degrees of central executive load was considerably weaker, reflecting the effects of increasing age and stronger executive function. Overall, the central executive load had a greater impact on children’s strategy choice than on their strategy execution. We can conclude that the complexity of the central executive load not only increases the intensity of resource competition, but also affects the development of strategy execution and strategy choice in childhood.

## Author Note

Project 31371048 supported by National Natural Science Foundation of China, project ZR2010CM059 supported by Shandong Province Natural Science Foundation, and the Key Subject Funds of Shandong Province, P. R. China (2011–2015).
